# The Metabolome in Different Sites of Gut Tract Regulates the Meat Quality of Longissimus Dorsi Muscle

**DOI:** 10.3390/ani15162399

**Published:** 2025-08-15

**Authors:** Binlong Chen, Tingting Zheng, Xue Bai, Weihua Chang, Yi Zhang, Shizhong Yang, Hao Li, Diyan Li, Tao Wang

**Affiliations:** 1 College of Animal Science, Xichang University, Xichang 615000, China; changweihua112@163.com (W.C.); zhangyis707@163.com (Y.Z.); xcysz1565@163.com (S.Y.); lihao03260235@163.com (H.L.); 2 Sichuan Wildlife Rehabilitation and Breeding Research Center, Key Laboratory of Southwest China Wildlife 8 Resources Conservation (Ministry of Education), China West Normal University, Nanchong 637009, China; ztt12172024@163.com; 3 School of Basic Medical Sciences, Chengdu University, Chengdu 610106, China; 18792972819@163.com; 4 School of Pharmacy, Chengdu University, Chengdu 610106, China; lidiyan@cdu.edu.cn

**Keywords:** gut, metabolites, integrated metabolomics and transcriptomics, longissimus dorsi muscle, meat quality

## Abstract

Meat quality is influenced by genetic, nutritional, and microbial factors, with increasing attention on the role of gut-derived metabolites. In this study, we conducted untargeted metabolomics of 10 gut tract sites and RNA sequencing (RNA-seq) of longissimus dorsi muscles in Meigu goats and Liangshan black sheep raised under standardized conditions. Results showed that goat muscle contained significantly higher levels of essential amino acids (e.g., methionine) and specific fatty acids (e.g., C18:3_N6, C20:4_N6), suggesting improved nutritional quality. Transcriptomic analysis identified 3133 differentially expressed genes (DEGs), among which *ADCY1* and *SLC38A4* were upregulated in goats and strongly associated with meat traits. Using integrative correlation analysis, we uncovered 17 genes and 19 gut metabolites that were significantly correlated with more than eight meat quality parameters across multiple gut sites. Notably, these metabolites included bioactive compounds such as L-tyrosine ethyl ester and pelargonidin 3-O-glucoside, while genes were enriched in pathways related to amino acid transport, cAMP signaling, and muscle development. Together, these findings highlight a potential gut–muscle axis involving metabolite-mediated modulation of muscle gene expression, contributing to breed-specific differences in meat composition and quality. This study provides a valuable framework for improving ruminant meat quality through integrative multi-omics analysis.

## Simple Summary

Sheep and goats are two important livestock species with distinct meat characteristics. In this study, we examined how metabolites in ten gut regions influence gene expression and meat quality in the longissimus dorsi muscle of Meigu goats and Liangshan black sheep. We found that goat meat contained higher levels of essential amino acids and certain n-6 polyunsaturated fatty acids, which may contribute to its nutritional density and flavor development. In contrast, sheep meat showed higher levels of n-3 fatty acids, which are known for their health-promoting properties. Transcriptomic analysis revealed differences in muscle gene expression between the two species, with several genes (e.g., *SLC38A4*, *ADCY1*) associated with amino acid transport and lipid metabolism. Our findings provide insight into species-specific mechanisms shaping meat quality and suggest opportunities to optimize meat production through targeted feeding or breeding strategies adapted to each species.

## 1. Introduction

Sheep and goats are valued livestock species renowned for their meat production, significantly contributing to the meat industry in the Liangshan Yi Autonomous Prefecture. Stable growth in China’s sheep meat industry will enhance the income levels of farmers and herdsmen, thereby ensuring the economic stability of ethnic minority areas [1]. The Liangshan black sheep and Meigu black goat are recognized as National Geographic Indication products. The Liangshan black sheep ranks among the top 10 excellent germplasm resources of Chinese livestock and poultry. This rare black sheep, newly discovered in Butuo County [2], is referred to as the “black elf” by herdsmen. This breed demonstrates resistance to rough feeding and exhibits significantly higher production performance compared to local goats; adult Meigu black goat bucks can attain approximately 45 kg of weight. The breed also shows outstanding reproductive performance: males are sexually active year-round, and does can produce two litters per year, yielding at least three kids annually [3,4]. Adult male Liangshan black sheep weigh approximately 98 kg. Each Liangshan ewe is capable of two lambings per year, with each litter consisting of a single lamb [5].

Meat quality, a crucial aspect of livestock production, is influenced by various factors, including genetics, nutrition, management practices, and gut microbiota composition [6]. For consumers, meat quality has always been important, particularly in the twenty-first century [7]. With the rapid development of animal husbandry, concerns regarding the animal microbiome and metabolome have emerged, encompassing issues such as low feed conversion efficiency, nitrogen utilization efficiency, meat quality, and high methane emissions [8,9,10]. To produce high-quality meat, we must understand the characteristics of meat quality traits and the factors that control them. Previous studies have already demonstrated that Meigu black goats have higher amino acid levels and lower cholesterol content than Liangshan sheep [11].

The term “metabolome” refers to the complete collection of metabolites present in a biological system [12,13]. Metabolites are small molecules, typically less than 1500 Daltons, that serve as intermediates or end products of cellular metabolism [14]. These metabolites can be produced by the host organism or derived from external sources such as diet, microbes, or xenobiotics [15]. Researchers have effectively utilized the study of the metabolome to establish connections between observable traits (phenotypes) and an organism’s genetic makeup (genotypes) [16]. Growing interest in fecal metabolome analyses stems from the fact that feces represent the ultimate end product of the digestion processes occurring in the gut [17,18]. The microbial community residing in the animal gut is rich in metabolic activity and plays a critical role in host physiology and health [17]. The extensive and diverse repertoire of bacterial metabolic functions complements the metabolic capacities of the host, enabling it to break down otherwise indigestible carbohydrates and synthesize beneficial vitamins [19]. Moreover, microbial metabolites promote gut homeostasis and influence the development and function of the host’s immune system [20]. Recent studies have demonstrated that the gut tract metabolome relates to the meat quality of chicken [21], sheep [22], and pigs [23]. Thus, among the factors affecting meat quality, the gut tract metabolome has emerged as a significant contributor to shaping meat quality attributes. In particular, the composition of gut metabolites at different sites can profoundly impact the composition, flavor, and overall quality of meat derived from animals.

Glutamic acid is an important amino acid that enhances the palatability of meat [24]. The content of long-chain fatty acids affects meat tenderness; for example, higher levels of palmitic acid (16:0) and stearic acid (18:0) result in greater shear force and tougher meat [25]. Certain metabolites, such as fatty acids, glycogen, and amino acids, contribute to meat flavor production through the Maillard reaction during heat treatment [26]. Flavor is one of the most critical quality characteristics of meat products and significantly influences overall consumer acceptability [27].

The longissimus dorsi muscle, located in the loin of livestock animals, is highly valued for its tenderness, juiciness, and flavor. Understanding how metabolites present in different gut tract sites impact the quality of the longissimus dorsi muscle is essential for optimizing meat production practices and ensuring consumer satisfaction. Both Liangshan black sheep and Meigu black goats are listed as China Geographical Indication products and represent two iconic local breeds in southwestern Sichuan. Raised at the same altitude and under identical diets and management regimes, they nonetheless exhibit distinct carcass traits and meat qualities that cater to different economic preferences, providing an ideal comparative model for elucidating the gene–gut metabolism–meat quality axis. Therefore, this study explored the differential metabolites (DMs) in ten gut sites and the differentially expressed genes (DEGs) of the longissimus dorsi muscle between Liangshan black sheep and Meigu goats. We observed key genes regulating important DMs and meat quality (including amino acids, fatty acids, and flavor) in these two breeds through integrated metabolomic and transcriptomic analyses.

## 2. Materials and Methods

### 2.1. Sample Collection

**Figure 1:**
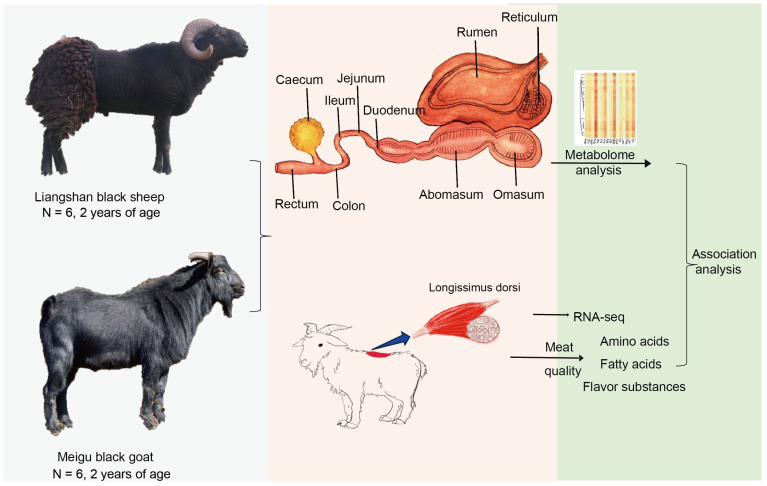
Schematic diagram of the procedure used to generate the gut metabolites, RNA-seq, and meat quality data (* *p* < 0.05).

All sheep and goats used in this study originated from the Liangshan Yi Autonomous Prefecture, Sichuan Province. The animals were approximately two years old, all male, and maintained under identical husbandry and management conditions, exhibiting comparable body weight, appearance, and health status. A total of six sheep and six goats were selected for sample collection. Both breeds are raised under intensive management and receive a daily ration consisting of green forage (natural or cultivated grasses and crop residues), concentrating supplements (including trace minerals and concentrates), and hay. All surgical procedures were performed under sodium pentobarbital anesthesia, and every effort was made to minimize animal suffering. The longissimus dorsi muscle was harvested from each animal and processed in six biological replicates to ensure analytical reproducibility (Figure 1). Additionally, we collected gut content from 10 sites (omasum, ileum, colon, jejunum, rumen, cecum, duodenum, reticulum, rectum, and abomasum) from these 12 individuals for metabolome analysis. All samples were promptly snap-frozen in liquid nitrogen, transported to the laboratory, and stored at −80 °C until further analysis. We managed the sheep and goats according to the guidelines of the Institutional Animal Care and Use Committee of Xichang University, under permit number XCU-20230708.

### 2.2. Amino Acid Content

Sample Hydrolysis: Accurately weigh and homogenize approximately 0.1000 g of the sample. Add 5 mL of 6 mol/L hydrochloric acid solution, seal the lid tightly, and vortex vigorously for 1 min to ensure thorough mixing. Purge the mixture with nitrogen, seal it with film, and place it in a pre-heated oven at 110 °C for a 24 h hydrolysis reaction.

Derivatization: After hydrolysis, cool the sample to room temperature. Add 5 mL of 6 mol/L sodium hydroxide solution, seal the lid tightly, and vortex vigorously for 1 min to neutralize the solution. Centrifuge the mixture at 5000 rpm for 10 min. Transfer 0.5 mL of the supernatant to a 5 mL brown centrifuge tube. Add 0.5 mL of 0.5 mol/L sodium bicarbonate solution with pH 9.0, followed by 0.5 mL of DNFB solution. Seal tightly, vortex for 1 min, cover with film, and place in a pre-heated water bath at 60 °C for 60 min, ensuring light protection. After the reaction, cool the mixture to room temperature. Adjust the volume to 5 mL with phosphate buffer at pH 7.0, and vortex for 1 min. Allow the solution to stand in the dark for 15 min before filtering 1 mL through a 0.22 μm membrane for analysis.

Test Conditions: Analyze the samples using an Agilent HPLC-1100 instrument equipped with a VWD detector (Agilent Technologies, Inc., Santa Clara, CA, USA). Use an Agilent C18 chromatographic column(Agilent Technologies, Inc., Santa Clara, CA, USA) (4.6 × 250 mm, 0.5 μm) with a column temperature set at 38 °C. Maintain a flow rate of 1 mL/min and an injection volume of 20 µL. Set the wavelength to 360 nm, and use the following mobile phase: A: 1 mol/L sodium acetate solution at pH 5.3; B:methanol = 1:1 (V), employing an isocratic elution method.

### 2.3. Fatty Acid Content

Sample Extraction and Purification: We weighed 0.50 g of the homogenized sample and added 5 mL of extraction solvent. The mixture was vortex-mixed at high speed for 1 min, then placed in a 50 °C water bath for 90 min of ultrasonic extraction. After centrifugation at 4000 rpm, we transferred the supernatant to another tube, repeated the extraction, and combined both extracts. We added 0.5 g of anhydrous sodium sulfate for drying and vortex-mixed for 30 s. Following centrifugation at 4000 rpm for 10 min, we transferred the supernatant to another tube and dried it with nitrogen at 50 °C to obtain the fat. Next, we added 5 mL of n-hexane and 3 mL of methanol-potassium hydroxide, vortex-mixed for 60 min, and methylated the solution in an oven at 30 °C. Finally, we concentrated the supernatant to 0.5 mL and filtered it through a 0.22 μm membrane for analysis.

Test Conditions: We used an Agilent GC6890 to analyze fatty acid content (Agilent Technologies, Santa Clara, CA, USA). The chromatographic column was DB-FFAP (60 × 0.25 mm) with a flame ionization detector (FID). The injection volume was 1 μL, with the detector temperature set at 280 °C and the injector temperature at 250 °C. The flow rate was 1 mL/min, and the column oven operated isothermally at 180 °C. Carrier gas flow rates were Hydrogen:Air:Nitrogen = 40:400:40 mL/min, with a split mode of 50:1.

### 2.4. Flavor Content

#### 2.4.1. Solid-Phase Microextraction

We analyzed the flavor content of each meat sample using an Agilent 8860 5977B (Agilent Technologies, Santa Clara, CA, USA). We placed 4 g of each muscle sample into a 20 mL headspace bottle and added 0.8 g of NaCl. After mixing, we heated the headspace bottle containing the sample in a constant-temperature water bath at 60 °C for 25 min, allowing the volatile compounds to reach equilibrium. We then inserted the extraction needle into the headspace bottle to adsorb the volatile gas. The extraction time was set to 2400 s using a 75 μm PDMS/DVB/CAR extraction head. We inserted the needle to a depth of 15 mm, with a coating extension length of 12 mm. The stirring speed was maintained at 300 r/min for 600 s. The analytical temperature was 270 °C, with an analysis duration of 300 s and an insertion depth of 20 mm.

#### 2.4.2. Gas Chromatography Conditions

DB-5ms Capillary Column Parameters: We used a DB-5ms capillary column (Agilent Technologies, Santa Clara, CA, USA) (30 m × 0.25 mm × 0.25 μm). The column temperature was programmed to start at 40 °C, held for 2 min, then increased at a rate of 6 °C/min to 160 °C, held for 0 min, followed by a ramp-up at 10 °C/min to 250 °C, and held for 10 min. The carrier gas flow rate was set to 1.4 mL/min. We employed a mass selective detector (MSD) with helium as the carrier gas at a flow rate of 40 mL/min. The split injection ratio was 5:1, and the septum purge flow rate was 3 mL/min. The injection port temperature was maintained at 270 °C.

Ion Source Parameters: We utilized an electron ionization (EI) source with an electron energy of 70 eV. The ion source temperature was set to 230 °C, while the quadrupole temperature was maintained at 150 °C. The transfer line temperature was 280 °C. We operated in scanning mode (Scan/SIM) with a mass range for scanning of 35–550 and a solvent delay of 1 min.

### 2.5. Total RNA-Seq and Data Analysis

We extracted total RNA from muscle tissue samples using the Qiagen RNeasy kit (Qiagen, Valencia, CA, USA). We generated sequencing libraries from 12 samples with the NEBNext Ultra RNA Library Prep Kit for Illumina (New England Biolabs, Ipswich, MA, USA) (NEB, USA, Catalog #: E7530L). Following the manufacturer’s recommendations, we isolated mRNA using poly-T oligo-attached magnetic beads. We performed paired-end sequencing (2 × 150 bp) on the DNBSEQ-T7 platform. The resulting clean data were then aligned to the sheep and goat reference genomes (ARS-UI_Ramb_v2.0 [28] and ARS1 [29]) using STAR (v2.7.6a) [30]. We assembled the aligned reads with StringTie v1.3.3 and constructed transcripts using Cufflinks 2.0.2 software [31,32]. We quantified expression levels of transcripts as transcripts per million (TPM) values using StringTie (v1.3.3). We defined transcripts with TPM ≥ 0.5 in at least two biological replicates as expressed protein-coding genes (PCGs). We conducted differential expression analysis of PCGs using DESeq2 (v1.34.0) [33]. To assess the biological significance of differentially expressed genes, we utilized Metascape (v3.5.20250701) for gene ontology enrichments of each cluster [34,35].

### 2.6. Gut Metabolite Analysis

#### 2.6.1. Metabolite Extraction

We added 50 mg of gut contents from each sample to a 2 mL centrifuge tube, along with a 6 mm diameter grinding bead. For metabolite extraction, we used 400 μL of extraction solution (methanol:water = 4:1 (v)) containing 0.02 GUtmg/mL of the internal standard, L-2-chlorophenylalanine. We ground the samples using the Wonbio-96c frozen tissue grinder (Shanghai Wanbo Biotechnology Co., Ltd., Shanghai, China) for 6 min at −10 °C and 50 Hz, followed by low-temperature ultrasonic extraction for 30 min at 5 °C and 40 kHz. After leaving the samples at −20 °C for 30 min, we centrifuged them for 15 min at 4 °C and 13,000× *g*. We then transferred the supernatant to the injection vial for LC-MS/MS analysis.

#### 2.6.2. Quality Control Sample

To ensure system conditioning and quality control, we prepared a pooled quality control (QC) sample by mixing equal volumes of all samples. We disposed of and tested the QC samples in the same manner as the analytical samples. This approach allowed the QC sample to represent the entire sample set, which was injected at regular intervals (every 5–15 samples) to monitor the stability of the analysis.

#### 2.6.3. UHPLC-MS/MS Analysis

We conducted the LC-MS/MS analysis using a Thermo UHPLC-Q Exactive HF-X system equipped with an ACQUITY HSS T3 column (100 mm × 2.1 mm i.d., 1.8 μm; Waters, Milford, MA, USA) at Majorbio Bio-Pharm Technology Co., Ltd. (Shanghai, China). The mobile phases included 0.1% formic acid in water (95:5, *v*/*v*) (solvent A) and 0.1% formic acid in acetonitrile: isopropanol (47.5:47.5, *v*/*v*) (solvent B). We maintained a flow rate of 0.40 mL/min and a column temperature of 40 °C, with an injection volume of 3 μL.

#### 2.6.4. MS Conditions

We collected mass spectrometric data using a Thermo UHPLC-Q Exactive HF-X Mass Spectrometer (Thermo Fisher Scientific, Bremen, Germany) equipped with an electrospray ionization (ESI) source, operating in both positive and negative modes. The optimal settings included a source temperature of 425 °C, a sheath gas flow rate of 50 arb, and an auxiliary gas flow rate of 13 arb. The ion-spray voltage floating (ISVF) was set at −3500 V for negative mode and +3500 V for positive mode. We applied a normalized collision energy of 20-40-60 V for MS/MS. Full MS resolution was set to 60,000, while MS/MS resolution was 7500. Data acquisition occurred in Data-Dependent Acquisition (DDA) mode, covering a mass range of 70–1050 *m*/*z*.

#### 2.6.5. Data Analysis

We performed the pretreatment of LC/MS raw data using Progenesis QI (v2.0) (Waters Corporation, Milford, CT, USA) software and exported a three-dimensional data matrix in CSV format. This matrix included sample information, metabolite names, and mass spectral response intensities. We removed internal standard peaks and any known false positive peaks (including noise, column bleed, and derivatized reagent peaks) from the data matrix, while deredundizing and pooling the peaks. Simultaneously, we identified the metabolites by searching databases, including the Human Metabolome Database (HMDB, http://www.hmdb.ca/, accessed on 1 July 2024), Metlin (https://metlin.scripps.edu/, accessed on 5 July 2024), and Majorbio Database. The resulting data matrix was uploaded to the Majorbio cloud platform (https://cloud.majorbio.com, accessed on 5 July 2024) for further analysis.

First, we pre-processed the data matrix by retaining at least 80% of the metabolic features detected in any set of samples. For specific samples with metabolite levels below the lower limit of quantification, we estimated the minimum metabolite value and normalized each metabolic signature to the sum. To reduce errors caused by sample preparation and instrument instability, we normalized the response intensities of the sample mass spectrometry peaks using the sum normalization method, resulting in a normalized data matrix. Additionally, we excluded variables from QC samples with a relative standard deviation (RSD) greater than 30% and log10-transformed the data to obtain the final data matrix for subsequent analysis. Next, we employed the R package “ropls” (Version 1.6.2) to perform principal component analysis (PCA) and orthogonal partial least squares discriminant analysis (OPLS-DA), including a 7-cycle interactive validation to evaluate the stability of the model. We identified metabolites with a variable importance in projection (VIP) greater than 1 and a *p*-value less than 0.05 as significantly different metabolites based on the results from the OPLS-DA model and the *p*-value generated by Student’s *t*-test.

We mapped differential metabolites between the two groups to their biochemical pathways through metabolic enrichment and pathway analysis based on the Kyoto Encyclopedia of Genes and Genomes database (KEGG, http://www.genome.jp/kegg/, accessed on 25 June 2024). We classified these metabolites according to the pathways they participate in and the functions they perform. We conducted enrichment analysis to determine whether a group of metabolites appeared in a functional node. This process involved evolving the annotation analysis of individual metabolites into an analysis of a group of metabolites. (https://docs.scipy.org/doc/scipy/, accessed on 12 August 2025). To identify the most relevant biological pathways associated with the experimental treatments, we utilized the Python package (v1.11.4) “scipy.stats” (https://docs.scipy.org/doc/scipy/, accessed on 12 August 2025) to perform the enrichment analysis.

## 3. Results

### 3.1. Amino Acid and Fatty Acid Composition of Sheep and Goats

**Figure 2:**
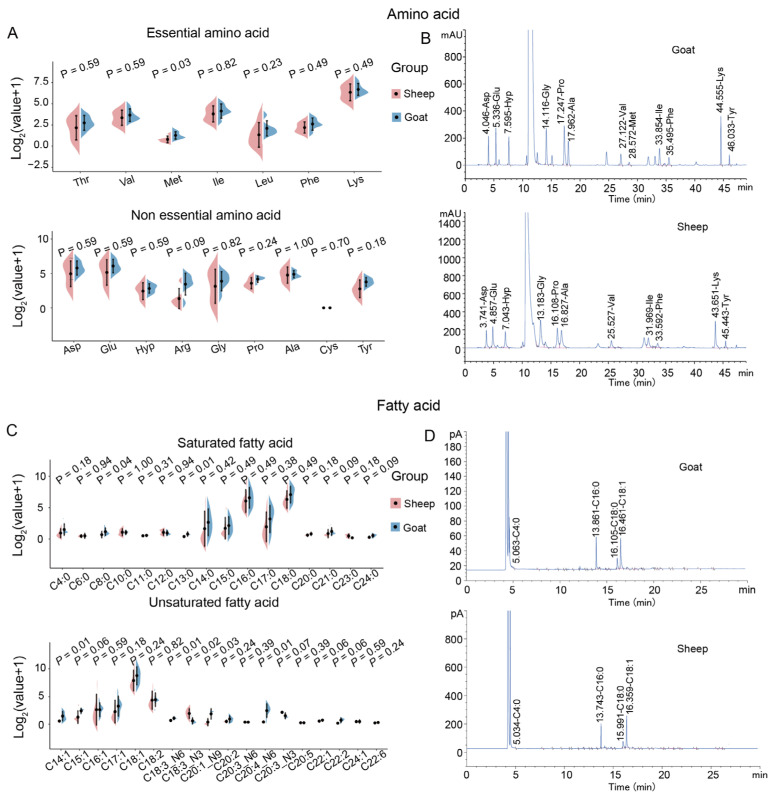
(**A**) Profiles of the essential and non-essential amino acid of the longissimus dorsi muscle from goat and sheep in µg/mL (*n* = 6). Thr, threonine; Val, valine; Met, methionine; Ile, isoleucine; Leu, leucine; Phe, phenylalanine; Lys, lysine; Asp, aspartic acid; Glu, glutamic acid; Hyp, hydroxyproline; Arg, L-arginine; Gly, glycine; Pro, proline; Ala, alanine; Cys, cystine; Tyr, tyrosine. (**B**) Examples showing detection of amino acid compositions in sheep and goats. (**C**) Violon plot showing saturated and unsaturated fatty acid profiles of longissimus dorsi muscles from goats and sheep (*n* = 6). (**D**) Examples showing detection of fatty acid compositions in sheep and goats.

We investigated the effect of gut metabolites and longissimus dorsi muscle gene expression on the meat quality of the longissimus dorsi muscle in goats and sheep. We performed metabolomic analyses of 10 gut sites, assessing meat quality and gene expression in the longissimus dorsi muscle of goats and sheep (Figure 1). The analyses revealed the amino acid and fatty acid profiles present in longissimus dorsi muscle samples from Liangshan black sheep and Meigu goats (Figure 1). Among the 16 amino acids (AAs), both essential and non-essential, Meigu goats consistently exhibited higher levels than Liangshan black sheep (Figure 2A,B). Notably, methionine (Met), an essential amino acid, was significantly higher in Meigu goats (*p* = 0.026) compared to Liangshan black sheep. Similarly, of the 34 fatty acids examined, only C13:0 (*p* = 0.01), C14:1 (*p* = 0.01), C18:3_N6 (*p* = 0.01), and C20:4_N6 (*p* = 0.01) were significantly higher in Meigu goats than in Liangshan black sheep (Figure 2C,D). Conversely, C18:3_N3 (*p* = 0.02) was significantly higher in sheep than in goats (Figure 2C).

### 3.2. Flavor Characteristics of Sheep and Goats

**Figure 3:**
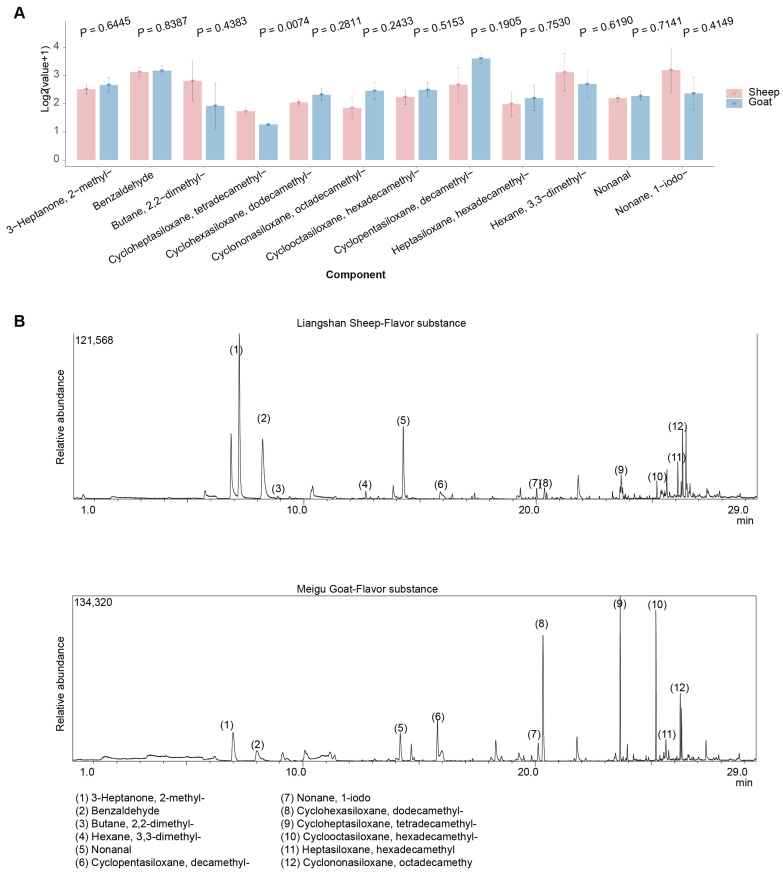
Flavor characteristics of sheep and goats. (**A**) Bar plot showing flavor profiles of the longissimus dorsi muscle from goats and sheep (*n* = 6). (**B**) Examples showing detection of flavor profiles in sheep (top) and goats (bottom).

Numerous methods exist for cooking mutton, each imparting distinct flavors to the product, including boiling, roasting, and frying [36]. Mutton is a multi-component complex system comprising proteins, lipids, water, carbohydrates, inorganic compounds, vitamins, and other constituents, which collectively contribute to its flavor [37,38]. Goat sausages generally have a tougher, more fibrous texture and are less juicy compared to sheep sausages [37]. In this study, we examined the flavor characteristics of the longissimus dorsi muscle in sheep and goats. Among the detected samples, only Cycloheptasiloxane, tetradecamethyl–exhibited a differentiated concentration between sheep and goats (Figure 3A). The three flavors with the highest average content across all samples were Cyclopentasiloxane, decamethyl- (8.62), Nonane, 1-iodo- (9.91), and Hexane, 3,3-dimethyl- (9.29) (Figure 3B).

### 3.3. Differentially Expressed Genes of Longissimus Dorsi Muscle in Sheep and Goats

To explore the mRNA expression patterns of the longissimus dorsi muscle in sheep and goats, we sequenced the transcriptomes of six sheep and six goats. We obtained a total of 270.2 million reads from the longissimus dorsi muscle of both sheep and goats. After filtering low-quality sequences, we selected 40.2 gigabases from sheep and 40.3 gigabases from goats for further analysis (Table S1).

**Figure 4:**
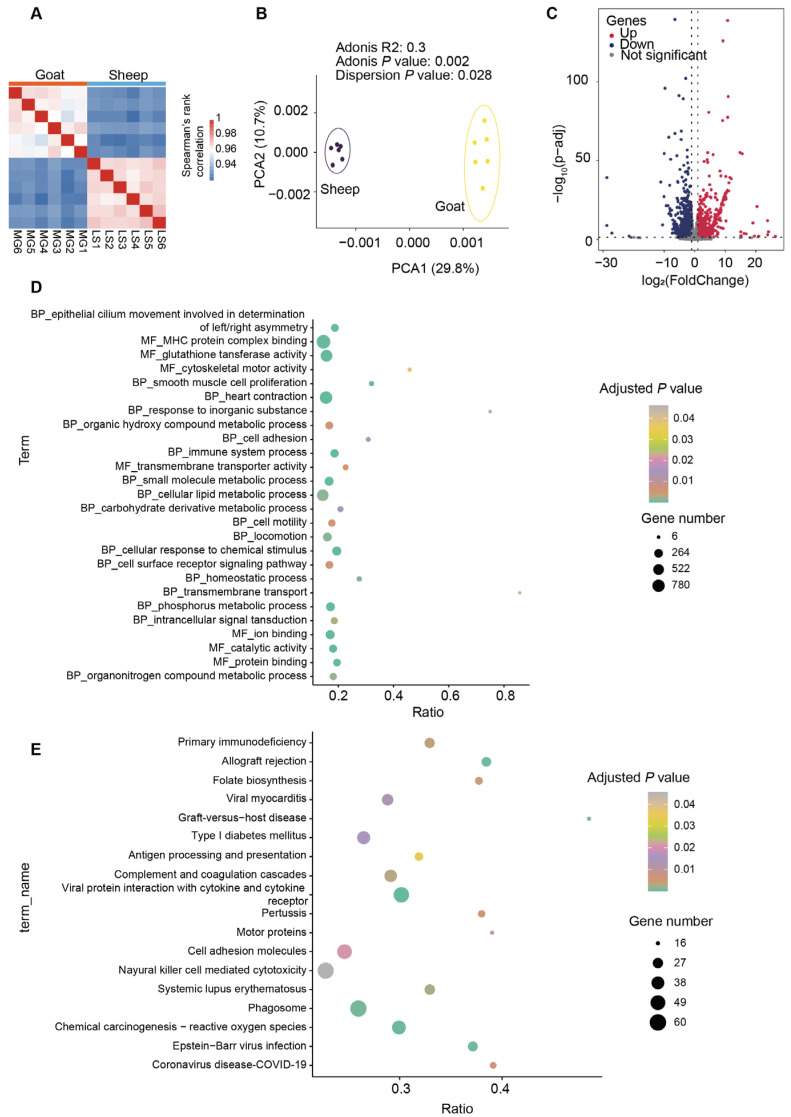
Differentially expressed genes and their functions in the longissimus dorsi muscle from sheep and goats. (**A**) Correlation analysis of mRNA expression across samples of sheep and goats. (**B**) Principal component plots with first and second dimensions shown. The fraction of the variance explained was 29.8% for eigenvector 1 and 10.7% for eigenvector 2. (**C**) Scatter plot of high-throughput sequencing data. Red (up-regulated genes) and blue (down-regulated genes) dots represent differentially expression genes, and grey dots represent similarly expressed genes. GO enrichment analysis (molecular function and biological process) (**D**) and KEGG (**E**) pathway enrichment of DEGs.

Next, we performed a correlation analysis to assess the variance of mRNA expression across replicates. All samples demonstrated high reproducibility within the same group, with Spearman correlation coefficients exceeding 0.94 (Figure 4A). The PCA revealed a significantly different expression profile between sheep and goats (Figure 4B). We used *p* < 0.05 and |log_2_ fold change| ≥ 1 to screen for DEGs. Our analysis identified 3133 significantly differentially expressed genes (Figure 4C). These DEGs primarily involved the following gene ontology (GO) terms: “cytoskeletal motor activity,” “glutathione transferase activity,” “MHC protein complex binding,” “smooth muscle cell proliferation,” and “epithelial cilium movement involved in determination of left/right asymmetry” (Figure 4D). Additionally, the DEGs were associated with the following KEGG pathways: “motor proteins,” “cell adhesion molecules,” “antigen processing and presentation,” and “primary immunodeficiency” (Figure 4E).

### 3.4. Gut Flora Metabolome Composition and Function of Sheep and Goats

**Figure 5:**
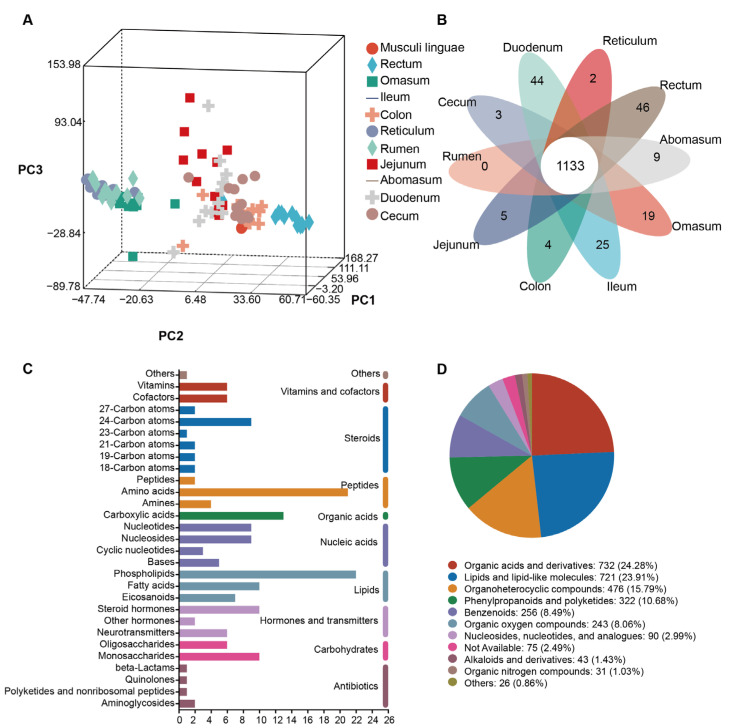
Composition and function of the gut flora metabolome. (**A**) Principal component analysis (PCA) of ten gut sites. (**B**) Venn diagram analysis revealing metabolites specific to each gut site or shared among all sites. (**C**) KEGG compound classifications for all metabolites. (**D**) HMDB compound classifications for all metabolites.

To explore the gut flora metabolome composition and function in sheep and goats at different sites, we analyzed the differences in metabolites across 10 gut sites (omasum, ileum, colon, jejunum, rumen, cecum, duodenum, reticulum, rectum, and abomasum) in Liangshan black sheep and Meigu goats using non-targeted metabolomic analysis with LC/MS. We identified a total of 1598 metabolites in positive mode and 1820 metabolites in negative mode. PCA revealed that the rectum site separated distinctly from the other sites (Figure 5A).

For the Venn analysis, we removed metabolites present in fewer than 50 samples. This analysis showed that the ten gut sites shared 1133 metabolites, with the rectum exhibiting the highest number of specific metabolites (46 metabolites) (Figure 5B).

We compared all identified metabolites against the KEGG and HMDB to obtain annotation information. Most of the KEGG compounds consisted of peptides, organic acids, and lipids (Figure 5C). In the HMDB annotation, the majority comprised organic acids and derivatives (732, 24.28%), lipids and lipid-like molecules (721, 23.91%), and organoheterocyclic compounds (476, 15.79%) (Figure 5D).

### 3.5. Differential Metabolites in Ten Gut Sites of Sheep and Goats

**Figure 6:**
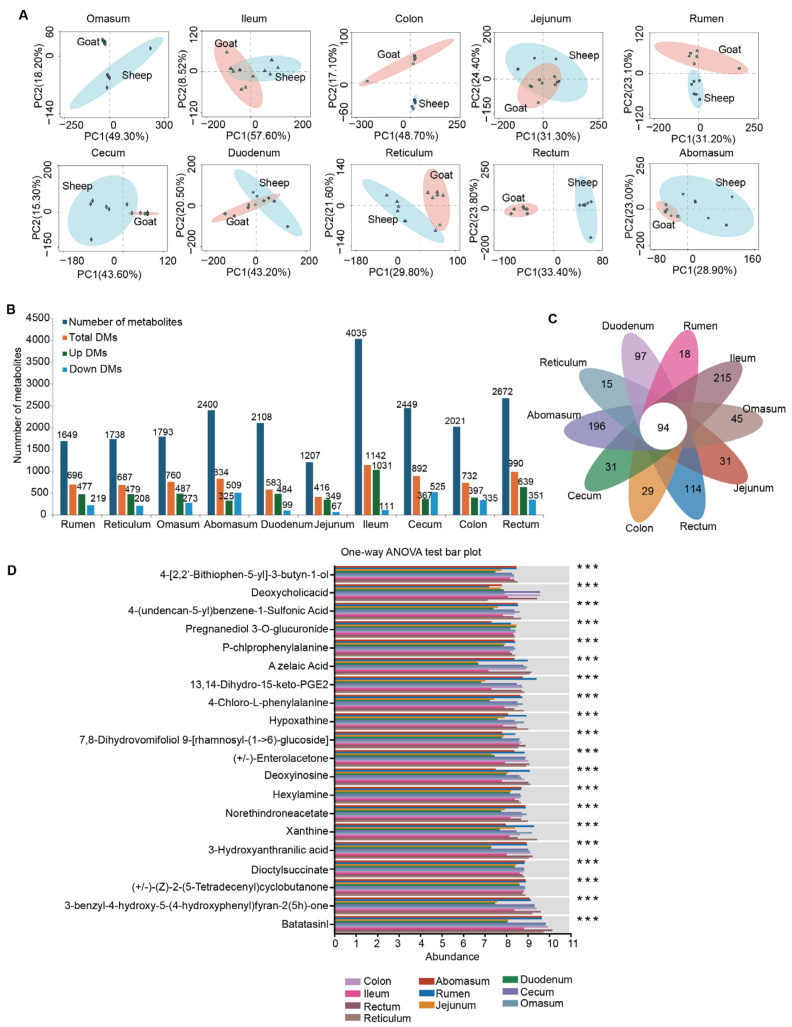
Differential metabolites in different gut sites of sheep and goats. (**A**) Principal component analysis of each gut site. (**B**) Number of differential metabolites (DMs) in each gut site. (**C**) Venn diagram analysis revealing DMs specific to each gut site or shared among all sites. (**D**) One-way ANOVA analysis showing DMs among ten gut sites (*** *p* < 0.001).

To explore the differences in metabolites at the same gut site between sheep and goats, we compared each site. By screening for DMs using criteria of *p* < 0.05 and VIP > 1, both PCA (Figure 6A) and differential metabolites analysis (Figure 6B) indicated that the metabolites in the duodenum and jejunum of goats and sheep were more similar than those in other sites, resulting in the least number of DMs. We identified 94 DMs shared among the ten gut sites based on comparisons between sheep and goats (Figure 6C). Next, we investigated the DMs across the ten gut sites using one-way ANOVA and post hoc tests. This analysis revealed 20 DMs that differed among the gut sites, including Batatasin I, 3-benzyl-4-hydroxy-5-(4-hydroxyphenyl)furan-2(5H)-one, (+/−)-(Z)-2-(5-tetradecenyl)cyclobutanone, dioctyl succinate, 3-hydroxyanthranilic acid, xanthine, hexylamine, deoxyinosine, (+/−)-enterolactone, 7,8-dihydrovomifoliol 9-[rhamnosyl-(1-6)-glucoside], hypoxanthine, and 4-chloro-L-phenylalanine, among others (Figure 6D).

### 3.6. Weighted Gene Co-Expression Network Analysis of Metabolites

**Figure 7:**
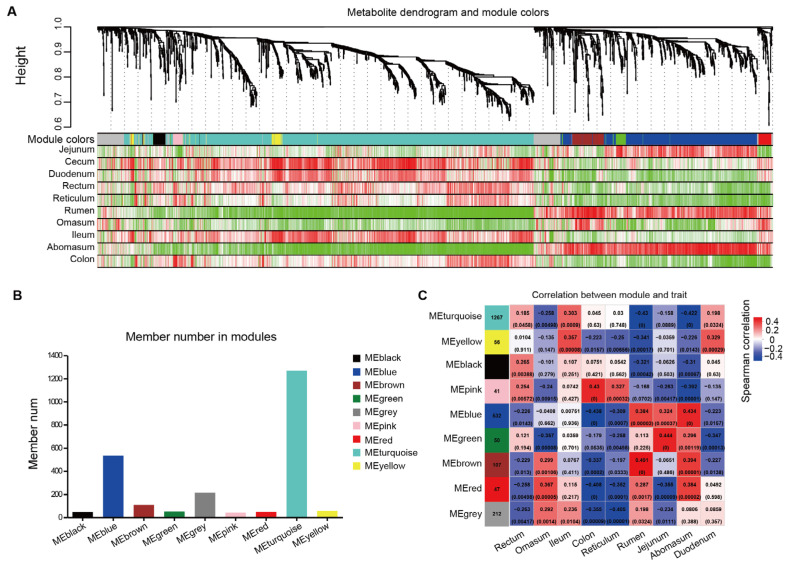
Weighted Gene Co-expression Network Analysis (WGCNA). (**A**) Clustering dendrogram of metabolites showing the metabolite hierarchical clustering tree (top), the module that metabolites belong to (middle), and the heat map of the correlation between metabolites and phenotypes in the modules (bottom). Each row represents each gut site, each column represents a metabolite in the module, and the color represents the correlation (red, positive correlation; green, negative correlation). (**B**) Number of metabolites identified in the WGCNA-detected modules. (**C**) Module–trait associations. In the heatmap, each row corresponds to a module eigengene (ME), and each column corresponds to a gut site. Each cell contains the corresponding correlation and *p*-value. The table is color-coded by correlation according to the color legend.

Weighted Gene Co-expression Network Analysis (WGCNA) constructs co-expression network relationships to cluster metabolites exhibiting similar expression patterns. By segmenting the network into modules based on similarity and associating these modules with phenotypes for functional analysis, we can gain insights into the overall condition of the modules. This process allows us to identify key hub metabolites within each module. Consequently, WGCNA is widely used to study the associations between diseases, phenotypes, and metabolites [39]. We constructed the weighted network using the Pearson correlation matrix, resulting in a final network consisting of nine modules (labeled by color), with sizes ranging from 47 to 1267 members (Figure 7A). The grey module, which grouped nodes with outlying profiles, was excluded from further analysis. The MEturquoise module contained the largest number of metabolites (1267 metabolites) (Figure 7B). Notably, metabolites in the MEturquoise module exhibited significant deficiencies in the three stomach sites (rumen, omasum, and abomasum) (Figure 7C).

### 3.7. Contribution of the Changes of Longissimus Dorsi Muscle Gene Expression and Gut Metabolites to the Shifts of Meat Quality

**Figure 8:**
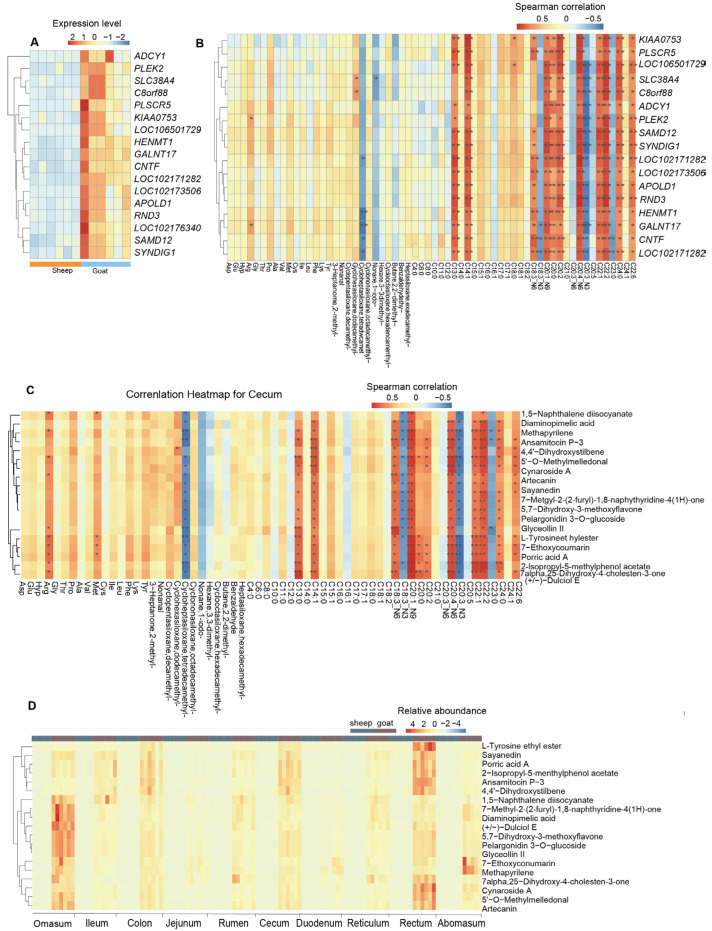
Correlations between gene expression, metabolite features, and longissimus dorsi muscle meat quality. (**A**) Gene expression profile of 17 genes correlated with more than 8 meat quality traits. (**B**) Correlations between differentially expressed genes and meat quality traits. (**C**) Correlations between shared differential metabolites and meat quality traits. DMs in at least 7 gut sites were found to be significantly correlated with one meat quality were retained. (**D**) Content heatmap of 19 differential metabolites correlated with longissimus dorsi muscle meat quality in ten gut sites. * *p* < 0.05; ** *p* < 0.01.

To explore how candidate DEGs and DMs contribute to the meat quality of the longissimus dorsi muscle, we focused on the 94 DMs shared among the ten gut sites based on comparisons between sheep and goats, as well as the DEGs identified in the longissimus dorsi muscle of both species. We calculated the correlations between the DMs and meat quality traits. Our analysis revealed that 17 genes—*ADCY1*, *PLEK2*, *SLC38A4*, *C8orf88*, *PLSCR5*, *KIAA0753*, *LOC106501729*, *HENMT1*, *GALNT17*, *CNTF*, *LOC102171282*, *LOC102173506*, *APOLD1*, *RND3*, *LOC102176340*, *SAMD12*, and *SYNDIG1* (Figure 8A)—were significantly associated with more than eight meat quality traits (Figure 8B). The expression levels of all 17 genes were higher in goats than in sheep (Figure 8A). Additionally, we identified 19 metabolites—2-isopropyl-5-methylphenol acetate, ansamitocin P-3, (+/−)-dulciol E, 1,5-naphthalene diisocyanate, 5′-O-methylmelledonal, cynaroside A, glyceollin II, L-tyrosine ethyl ester, methapyrilene, pelargonidin 3-O-glucoside, sayanedin, 4,4′-dihydroxystilbene, 5,7-dihydroxy-3-methoxyflavone, 7alpha,25-dihydroxy-4-cholesten-3-one, 7-ethoxycoumarin, 7-methyl-2-(2-furyl)-1,8-naphthyridine-4(1H)-one, artecanin, diaminopimelic acid, and porric acid A—which were significantly correlated with meat quality traits in at least seven gut sites (Figure 8C). Consistent with gene expression patterns, all 19 metabolites exhibited higher levels in goats than in sheep (Figure 8D).

## 4. Discussion

### 4.1. Goat Meat Exhibits Superior Nutritional and Flavor Profiles Compared to Sheep

The gastrointestinal tract plays a pivotal role in animal metabolism and significantly influences muscle quality by producing various metabolites. These gut-derived metabolites, including amino acids, short-chain fatty acids, and other bioactive compounds, are crucial for modulating muscle development, composition, and flavor, thereby directly impacting meat quality [6]. Understanding the complex interactions between these metabolites and muscle tissues is essential for enhancing livestock meat quality.

Globally, sheep and goats are significant commodities for providing meat for human consumption [40]. Their nutritional profile, which includes proteins, vitamins, and essential fatty acids, satisfies necessary dietary requirements [41]. In our comparison of the longissimus dorsi muscle quality traits between sheep and goats, we observed that the contents of amino acids and fatty acids exhibited a higher trend in goats. Additionally, Met (an essential amino acid), C13:0, C14:1, C18:3_N6, and C20:4_N6 were significantly more abundant in goats. Goat meat contains a low percentage of fat and serves as an excellent source of essential fatty acids [42].

### 4.2. Distinct Gut Metabolite Profiles Suggest Site-Specific Regulation of Muscle Traits

In this study, we identified 94 metabolites that exhibited significant differences across 10 distinct gut regions in both goats and sheep. Notably, certain amino acids, such as arginine, flavor-related compounds like cycloheptasiloxane, tetradecamethyl, and specific fatty acids including C13:0, C18:3_N6, C18:3_N3, C20:1_N9, C20:0, and C20:2 showed significant correlations with various metabolites across these gut regions. These findings indicate that gut metabolites substantially influence the profiles of amino acids, fatty acids, and flavor compounds in muscle tissues, which are key determinants of meat quality. The concentration of fatty acids in muscle tissue is primarily regulated by genes that encode specific metabolic enzymes or transcription factors involved in lipid metabolism [43]. This supports the hypothesis that gut regional metabolic activity contributes to systemic changes in muscle phenotype [21,22,23].

### 4.3. Muscle Gene Expression Is Coordinated with Gut-Derived Metabolite Signals

We then assessed the correlation between muscle gene expression and the content of amino acids, fatty acids, and flavor compounds. Transcriptomic analysis of longissimus dorsi muscle revealed 3133 differentially expressed genes between goats and sheep. As a result, we found that 17 genes significantly correlated with at least eight meat quality traits, all of which exhibited relatively higher expression in goats than in sheep. Among these, *ADCY1* and SLC38A4 were strongly associated with multiple meat quality traits. *ADCY1* emerged as a key gene potentially associated with pork meat characteristics [44]. Type 1 adenylyl cyclase (*ADCY1*) functions as an enzyme that generates cAMP, making it a crucial component of the cAMP signaling and regulatory pathways [45], and it has been linked to chest width [46]. Upregulation of *ADCY1* could enhance cAMP production, modulating protein kinase A (PKA) and downstream transcription factors such as CREB. These pathways have been linked to muscle differentiation, hypertrophy, and even growth hormone regulation [47,48]. This may explain the broader impact of *ADCY1* on multiple meat quality traits in goats, as cAMP signaling has pleiotropic roles in lipid metabolism, flavor precursor biosynthesis, and muscle contractile properties.

We found that the expression of *SLC38A4* significantly positively affects meat quality, particularly regarding fatty acid content. *SLC38A4*, a member of the solute carrier protein family (SLC), functions as a system A amino acid transporter [49,50]. It is also known as the neutral amino acid transporter 4 (SNAT4). Amino acids are essential for the survival and proliferation of rapidly dividing cells, including embryonic and cancer cells [51]. Consequently, these rapidly dividing cells consistently exhibit high levels of amino acid transporter expression [52]. Here, elevated expression of *SLC38A4* may enhance intracellular amino acid availability in muscle cells, activating mTORC1 signaling, which is essential for protein accretion and muscle fiber development [53]. This mechanism can directly contribute to the higher amino acid content and improved texture observed in goat meat. We conclude that the high expression of *SLC38A4* facilitates amino acid transport in muscle tissue, resulting in improved meat quality. These genes potentially link metabolite signals with downstream physiological changes in muscle.

### 4.4. Coordinated Action of Gut Metabolites and Muscle Genes Drives Meat Quality

By correlating 94 gut metabolites and 17 key genes with more than eight meat quality traits, we identified 19 metabolites, including L-tyrosine ethyl ester, pelargonidin 3-O-glucoside, and porric acid A, that were consistently elevated in goats. Some of these (e.g., polyphenols) are known antioxidants, possibly protecting muscle lipids from oxidation, thus improving flavor and shelf life [41]. The co-expression of these metabolites and genes (revealed via WGCNA) suggests a gut–muscle regulatory axis, whereby microbiota-derived or host-processed metabolites may act via systemic or paracrine signaling pathways.

The integration of WGCNA modules and transcriptomic correlation analyses suggests a regulatory link between gut metabolites and muscle gene expression. Notably, amino acids such as tyrosine and arginine, which were enriched in multiple gut sites in goats, positively correlated with higher expression of *SLC38A4* in muscle—a transporter facilitating amino acid uptake and activating downstream mTOR signaling [51]. Similarly, increased levels of lipid-related metabolites like pelargonidin 3-O-glucoside may impact cAMP production via adenylyl cyclase (*ADCY1*), which modulates transcriptional programs for lipid metabolism and cell differentiation [45]. While our findings are correlational, the strong module–trait and gene–metabolite associations imply a possible gut–muscle axis that warrants future mechanistic investigation.

However, despite these correlations, the precise molecular mechanisms through which gut metabolites regulate muscle quality traits remain to be fully elucidated. Recent studies have delved into the innate differences between different breeds through 3D genome and single-cell transcriptome analyses [54,55,56], shedding light on the genetic mechanisms underlying the innate difference of breeds. These studies have provided valuable insights into the genetic difference for breeding improvement. Further research is necessary to explore these interactions more deeply, potentially uncovering novel pathways that could enhance meat quality in ruminants.

### 4.5. Implications for Breeding, Conservation, and Local Meat Production

Our findings have important implications for both livestock breeding strategies and local breed preservation. The Meigu goat and Liangshan black sheep are recognized as China Geographical Indication products, traditionally raised under extensive or semi-intensive systems. The consistently higher levels of essential amino acids (e.g., methionine, valine), favorable fatty acid profiles (e.g., elevated n-6 PUFA like C20:4_n6), and bioactive flavor compounds in Meigu goats compared to sheep and other breeds highlight a distinct nutritional advantage not often observed in commercial goat lines. Moreover, transcriptomic analysis revealed significantly higher expression of *ADCY1* and *SLC38A4*, which have direct links to amino acid transport and anabolic signaling, suggesting these breeds may possess unique genetic mechanisms favoring muscle development and meat quality under natural feeding conditions. These breed-specific gene-metabolite-meat trait associations have not been reported in intensively selected commercial breeds such as Boer or Suffolk crosses, emphasizing the genomic value of local germplasm. From a production standpoint, these traits suggest that Meigu goats could serve as a valuable genetic reservoir for enhancing meat quality in goat breeding programs, particularly for regions where forage-based feeding predominates. Furthermore, maintaining the genetic diversity and adaptive metabolic profiles of these local breeds may support sustainable livestock production under environmental or dietary stress, offering long-term advantages over genetically uniform commercial lines. In this context, our study supports not only scientific understanding of the gut–muscle–meat axis but also contributes to evidence-based strategies for conserving and utilizing native livestock resources.

## 5. Conclusions

The study reveals that goat meat tends to have higher levels of essential amino acids and specific fatty acids compared to sheep, contributing to its superior nutritional profile. The differential gene expression, particularly the higher levels of *ADCY1* and *SLC38A4* in goats, suggests a role in improved amino acid transport and meat quality. Additionally, gut metabolites vary significantly between sheep and goats, potentially influencing meat characteristics. Further research is essential to understand the molecular mechanisms linking gut metabolites and muscle quality in ruminants.

## Data Availability

RNA-Seq data of sheep and goat the longissimus dorsi muscles have been deposited into the National Center for Biotechnology Information (NCBI) Sequence Read Archive (SRA) database (Experiments for SRP485567) under BioProject accession number PRJNA106782024.

## Supplementary Materials

The following supporting information can be downloaded at https://www.mdpi.com/article/10.3390/ani15162399/s1, Table S1: Data description of all RNA-seq samples.

## References

[1] Fu L., Shi J., Meng Q., Tang Z., Liu T., Zhang Q., Cheng S. (2024). Verification of Key Target Molecules for Intramuscular Fat Deposition and Screening of SNP Sites in Sheep from Small-Tail Han Sheep Breed and Its Cross with Suffolk. Int. J. Mol. Sci..

[2] Zhou M., Wang G., Chen M., Pang Q., Jiang S., Zeng J., Du D., Yang P., Wu W., Zhao H. (2021). Genetic diversity and population structure of sheep (Ovis aries) in Sichuan, China. PLoS ONE.

[3] Guo J., Tao H., Li P., Li L.I., Zhong T., Wang L., Ma J., Chen X., Song T., Zhang H. (2018). Whole-genome sequencing reveals selection signatures associated with important traits in six goat breeds. Sci. Rep..

[4] Weijie Y. (2022). Breeding Technology and Demonstration Promotion of Meigu goat. Livest. Sci..

[5] Quzhe E.J.W., Cuo P., Muqie B., Lei R., Zhangjia B., Shizhong Y., Amei J. (2024). Study on the Measurement of Body Weight, Body Size, and Feed Intake of Liangshan Black Sheep at Different Growth Stages. Sichuan Anim. Vet. Sci..

[6] Chen B., Li D., Leng D., Kui H., Bai X., Wang T. (2022). Gut microbiota and meat quality. Front. Microbiol..

[7] Joo S.T., Kim G.D., Hwang Y.H., Ryu Y.C. (2013). Control of fresh meat quality through manipulation of muscle fiber characteristics. Meat Sci..

[8] Nkrumah J.D., Okine E.K., Mathison G.W., Schmid K., Li C., Basarab J.A., Price M.A., Wang Z., Moore S.S. (2006). Relationships of feedlot feed efficiency, performance, and feeding behavior with metabolic rate, methane production, and energy partitioning in beef cattle. J. Anim. Sci..

[9] Kumar S., Dagar S.S., Puniya A.K., Upadhyay R.C. (2013). Changes in methane emission, rumen fermentation in response to diet and microbial interactions. Res. Vet. Sci..

[10] Gharechahi J., Vahidi M.F., Bahram M., Han J.L., Ding X.Z., Salekdeh G.H. (2021). Metagenomic analysis reveals a dynamic microbiome with diversified adaptive functions to utilize high lignocellulosic forages in the cattle rumen. ISME J..

[11] Leng D., Huang Z., Bai X., Wang T., Zhang Y., Chang W., Zhao W., Li D., Chen B. (2024). Gene expression profiles in specific skeletal muscles and meat quality characteristics of sheep and goats. Sci. Data.

[12] Lamichhane S., Sen P., Dickens A.M., Orešič M., Bertram H.C. (2018). Gut metabolome meets microbiome: A methodological perspective to understand the relationship between host and microbe. Methods.

[13] Lamichhane S., Yde C.C., Schmedes M.S., Jensen H.M., Meier S., Bertram H.C. (2015). Strategy for Nuclear-Magnetic-Resonance-Based Metabolomics of Human Feces. Anal. Chem..

[14] Oliver S.G., Winson M.K., Kell D.B., Baganz F. (1998). Systematic functional analysis of the yeast genome. Trends Biotechnol..

[15] Fiehn O. (2002). Metabolomics—The link between genotypes and phenotypes. Plant Mol. Biol..

[16] Macel M., Van Dam N.M., Keurentjes J.J. (2010). Metabolomics: The chemistry between ecology and genetics. Mol. Ecol. Resour..

[17] Muller E., Algavi Y.M., Borenstein E. (2022). The gut microbiome-metabolome dataset collection: A curated resource for integrative meta-analysis. npj Biofilms Microbiomes.

[18] Su Y., Ge Y., Xu Z., Zhang D., Li D. (2021). The digestive and reproductive tract microbiotas and their association with body weight in laying hens. Poult. Sci..

[19] Van Treuren W., Dodd D. (2020). Microbial Contribution to the Human Metabolome: Implications for Health and Disease. Annu. Rev. Pathol..

[20] Postler T.S., Ghosh S. (2017). Understanding the Holobiont: How Microbial Metabolites Affect Human Health and Shape the Immune System. Cell Metab..

[21] Zhou X., Zhang H., Li S., Jiang Y., Kang L., Deng J., Yang C., Zhao X., Zhao J., Jiang L. (2023). The effects of fermented feedstuff derived from Citri Sarcodactylis Fructus by-products on growth performance, intestinal digestive enzyme activity, nutrient utilization, meat quality, gut microbiota, and metabolites of broiler chicken. Front. Vet. Sci..

[22] Ma Y., Yang X., Hua G., Deng X., Xia T., Li X., Feng D., Deng X. (2022). Contribution of gut microbiomes and their metabolomes to the performance of Dorper and Tan sheep. Front. Microbiol..

[23] Goethals S., Rombouts C., Hemeryck L.Y., Van Meulebroek L., Van Hecke T., Vossen E., Van Camp J., De Smet S., Vanhaecke L. (2020). Untargeted Metabolomics to Reveal Red versus White Meat-Associated Gut Metabolites in a Prudent and Western Dietary Context. Mol. Nutr. Food Res..

[24] Rikimaru K., Takahashi H. (2010). Evaluation of the meat from Hinai-jidori chickens and broilers: Analysis of general biochemical components, free amino acids, inosine 5′-monophosphate, and fatty acids. J. Appl. Poult. Res..

[25] Zhao H., Chong J., Tang R., Li L., Xia J., Li D. (2018). Metabolomics investigation of dietary effects on flesh quality in grass carp (Ctenopharyngodon idellus). Gigascience.

[26] Li J., Tang C., Zhao Q., Yang Y., Li F., Qin Y., Liu X., Yue X., Zhang J. (2020). Integrated lipidomics and targeted metabolomics analyses reveal changes in flavor precursors in psoas major muscle of castrated lambs. Food Chem..

[27] Fu Y., Cao S., Yang L., Li Z. (2022). Flavor formation based on lipid in meat and meat products: A review. J. Food Biochem..

[28] Davenport K.M., Bickhart D.M., Worley K., Murali S.C., Salavati M., Clark E.L., Cockett N.E., Heaton M.P., Smith T.P., Murdoch B.M. (2022). An improved ovine reference genome assembly to facilitate in-depth functional annotation of the sheep genome. Gigascience.

[29] Bickhart D.M., Rosen B.D., Koren S., Sayre B.L., Hastie A.R., Chan S., Lee J., Lam E.T., Liachko I., Sullivan S.T. (2017). Single-molecule sequencing and chromatin conformation capture enable de novo reference assembly of the domestic goat genome. Nat. Genet..

[30] Dobin A., Davis C.A., Schlesinger F., Drenkow J., Zaleski C., Jha S., Batut P., Chaisson M., Gingeras T.R. (2013). STAR: Ultrafast universal RNA-seq aligner. Bioinformatics.

[31] Ghosh S., Chan C.K. (2016). Analysis of RNA-Seq Data Using TopHat and Cufflinks. Methods Mol. Biol..

[32] Pertea M., Pertea G.M., Antonescu C.M., Chang T.C., Mendell J.T., Salzberg S.L. (2015). StringTie enables improved reconstruction of a transcriptome from RNA-seq reads. Nat. Biotechnol..

[33] Love M.I., Huber W., Anders S. (2014). Moderated estimation of fold change and dispersion for RNA-seq data with DESeq2. Genome Biol..

[34] Cardoso-Moreira M., Halbert J., Valloton D., Velten B., Chen C., Shao Y., Liechti A., Ascenção K., Rummel C., Ovchinnikova S. (2019). Gene expression across mammalian organ development. Nature.

[35] Zhou Y., Zhou B., Pache L., Chang M., Khodabakhshi A.H., Tanaseichuk O., Benner C., Chanda S.K. (2019). Metascape provides a biologist-oriented resource for the analysis of systems-level datasets. Nat. Commun..

[36] Zhao B., Sun B., Wang S., Zhang Y., Zang M., Le W., Wang H., Wu Q. (2021). Effect of different cooking water on flavor characteristics of mutton soup. Food Sci. Nutr..

[37] Paulos K., Rodrigues S., Oliveira A.F., Leite A., Pereira E., Teixeira A. (2015). Sensory Characterization and Consumer Preference Mapping of Fresh Sausages Manufactured with Goat and Sheep Meat. J. Food Sci..

[38] Whitfield F.B. (1992). Volatiles from interactions of Maillard reactions and lipids. Crit. Rev. Food Sci. Nutr..

[39] Leng D., Yang M., Miao X., Huang Z., Li M., Liu J., Wang T., Li D., Feng C. (2024). Dynamic changes in the skin transcriptome for the melanin pigmentation in embryonic chickens. Poult. Sci..

[40] Watkins P.J., Jaborek J.R., Teng F., Day L., Castada H.Z., Baringer S., Wick M. (2021). Branched chain fatty acids in the flavour of sheep and goat milk and meat: A review. Small Rumin. Res..

[41] Domínguez R., Pateiro M., Gagaoua M., Barba F.J., Zhang W., Lorenzo J.M. (2019). A Comprehensive Review on Lipid Oxidation in Meat and Meat Products. Antioxidants.

[42] Aghwan Z.A., Alimon A.R., Goh Y.M., Nakyinsige K., Sazili A.Q. (2014). Fatty Acid Profiles of Supraspinatus, Longissimus lumborum and Semitendinosus Muscles and Serum in Kacang Goats Supplemented with Inorganic Selenium and Iodine. Asian-Australas. J. Anim. Sci..

[43] Zhang N., Teng Z., Qi Q., Hu G., Lian H., Gao T. (2020). Carcass traits, meat quality characteristics, and lipid metabolism-related gene expression pattern of Yaoshan white goats raised in traditional extensive production system: Effects of slaughter age and meat cuts. Small Rumin. Res..

[44] Wang K., Wu P., Wang S., Ji X., Chen D., Xiao W., Gu Y., Zeng Y., Xu X., Tang G. (2022). Differential DNA methylation analysis reveals key genes in Chinese Qingyu and Landrace pigs. Genome.

[45] Khannpnavar B., Mehta V., Qi C., Korkhov V. (2020). Structure and function of adenylyl cyclases, key enzymes in cellular signaling. Curr. Opin. Struct. Biol..

[46] Liu M., Woodward-Greene J., Kang X., Pan M.G., Rosen B., Van Tassell C.P., Chen H., Liu G.E. (2020). Genome-wide CNV analysis revealed variants associated with growth traits in African indigenous goats. Genomics.

[47] Yarwood S.J., Kilgour E., Anderson N.G. (1998). Cyclic AMP potentiates growth hormone-dependent differentiation of 3T3-F442A preadipocytes: Possible involvement of the transcription factor CREB. Mol. Cell Endocrinol..

[48] Harwood J.P., Grewe C., Aguilera G. (1984). Actions of growth hormone-releasing factor and somatostatin on adenylate cyclase and growth hormone release in rat anterior pituitary. Mol. Cell Endocrinol..

[49] Shi Q., Padmanabhan R., Villegas C.J., Gu S., Jiang J.X. (2011). Membrane topological structure of neutral system N/Aamino acid transporter 4 (SNAT4) protein. J. Biol. Chem..

[50] Kondou H., Kawai M., Tachikawa K., Kimoto A., Yamagata M., Koinuma T., Yamazaki M., Nakayama M., Mushiake S., Ozono K. (2013). Sodium-coupled neutral amino acid transporter 4 functions as a regulator of protein synthesis during liver development. Hepatol. Res..

[51] Bröer S. (2014). The SLC38 family of sodium-amino acid co-transporters. Pflugers Arch..

[52] Pizzagalli M.D., Bensimon A., Superti-Furga G. (2021). A guide to plasma membrane solute carrier proteins. FEBS J..

[53] Kim J., Dominguez Gutierrez G., Xin Y., Cavino K., Sung B., Sipos B., Kloeppel G., Gromada J., Okamoto H. (2019). Increased SLC38A4 Amino Acid Transporter Expression in Human Pancreatic α-Cells After Glucagon Receptor Inhibition. Endocrinology.

[54] Wang T., Leng D., Cai Z., Chen B., Li J., Kui H., Li D., Li Z. (2024). Insights into left-right asymmetric development of chicken ovary at the single-cell level. J. Genet. Genom..

[55] Li J., Lin Y., Li D., He M., Kui H., Bai J., Chen Z., Gou Y., Zhang J., Wang T. (2024). Building Haplotype-Resolved 3D Genome Maps of Chicken Skeletal Muscle. Adv. Sci..

[56] Leng D., Zeng B., Wang T., Chen B.L., Li D.Y., Li Z.J. (2024). Single nucleus/cell RNA-seq of the chicken hypothalamic-pituitary-ovarian axis offers new insights into the molecular regulatory mechanisms of ovarian development. Zool. Res..

